# The Relationship Between Dopamine Neurotransmitter Dynamics and the Blood-Oxygen-Level-Dependent (BOLD) Signal: A Review of Pharmacological Functional Magnetic Resonance Imaging

**DOI:** 10.3389/fnins.2018.00238

**Published:** 2018-04-10

**Authors:** Tyler J. Bruinsma, Vidur V. Sarma, Yoonbae Oh, Dong Pyo Jang, Su-Youne Chang, Greg A. Worrell, Val J. Lowe, Hang Joon Jo, Hoon-Ki Min

**Affiliations:** ^1^Department of Radiology, College of Medicine, Mayo Clinic, Rochester, MN, United States; ^2^Department of Pharmaceutics and Brain Barriers Research Center, College of Pharmacy, University of Minnesota, Minneapolis, MN, United States; ^3^Department of Biomedical Engineering, Hanyang University, Seoul, South Korea; ^4^Department of Neurologic Surgery, College of Medicine, Mayo Clinic, Rochester, MN, United States; ^5^Departments of Physiology and Biomedical Engineering, Mayo Clinic, Rochester, MN, United States; ^6^Department of Neurology, College of Medicine, Mayo Clinic, Rochester, MN, United States

**Keywords:** dopamine, BOLD, fMRI, pharma-fMRI, fast-scan cyclic voltammetry

## Abstract

Functional magnetic resonance imaging (fMRI) is widely used in investigations of normal cognition and brain disease and in various clinical applications. Pharmacological fMRI (pharma-fMRI) is a relatively new application, which is being used to elucidate the effects and mechanisms of pharmacological modulation of brain activity. Characterizing the effects of neuropharmacological agents on regional brain activity using fMRI is challenging because drugs modulate neuronal function in a wide variety of ways, including through receptor agonist, antagonist, and neurotransmitter reuptake blocker events. Here we review current knowledge on neurotransmitter-mediated blood-oxygen-level dependent (BOLD) fMRI mechanisms as well as recently updated methodologies aimed at more fully describing the effects of neuropharmacologic agents on the BOLD signal. We limit our discussion to dopaminergic signaling as a useful lens through which to analyze and interpret neurochemical-mediated changes in the hemodynamic BOLD response. We also discuss the need for future studies that use multi-modal approaches to expand the understanding and application of pharma-fMRI.

## Introduction

Positron emission tomography (PET) and functional magnetic resonance imaging (fMRI) are used in both basic and clinical research as an indicator of brain health, structurally and dynamically. In fMRI the most common measurement for evaluating changes in functional brain activity is the blood-oxygen-level dependent (BOLD) signal (Ogawa et al., 1990; Bandettini et al., 1992), and in PET it is the cerebral metabolic rate for glucose (CMRglc) (Phelps et al., 1979). Both depend on mapping energy expenditure during pre- and postsynaptic neuronal signaling, events that lead to a rapid need for oxygen and glucose from the vascular system (Attwell and Laughlin, 2001).

In fMRI, BOLD changes have been associated with local field potentials that comprise pre- and post-synaptic signals from synapses and dendrites (Logothetis et al., 2001; see Heeger and Ress, 2002 for full review on the association between fMRI and neuronal activity). A quantitative relationship between the cycling of certain neurotransmitters (e.g., glutamate) and the BOLD signal has been well defined. This relationship is considered a major factor in the BOLD signal (Shulman and Rothman, 1998; Magistretti et al., 1999; Rothman et al., 1999) and is reliant on a tightly coupled relationship between synaptic activity and glucose uptake (Pellerin and Magistretti, 1994; Takahashi et al., 1995). The control of blood flow in the brain by neurons and astrocytes in glutamatergic transmitter systems has been reviewed in a greater detail by (Attwell et al., 2010).

In this mini review, we focus on the dopamine (DA) neurotransmitter-signaling pathway and include in our discussion current theories on how DA affects hemodynamic change and the potential of multi-modal methodological approaches to more comprehensively describe dopaminergic-related hemodynamic mechanisms.

## Pharmacological fMRI (pharma-fMRI)

Neuroimaging has afforded new opportunities to assess the effects of drug interventions in the human brain, including serial, single-subject longitudinal studies. However, because pharmaceutical agents modulate neuronal activities in a variety of ways, imaging studies of the effects of neurochemical induced changes remain challenging. Receptor agonist, antagonist, and neurotransmitter reuptake blocker events translate to diverse and often poorly understood alterations in mental and physical states. PET has been considered the gold standard for molecular imaging because it can assess the binding of radioactive tracers to selected receptor targets and can provide quantitative information on the binding, receptor occupancy, and regional brain distribution of targeted pharmaceuticals (Nguyen et al., 2000; Jenkins, 2012; Lu et al., 2014).

More recently, pharmacological fMRI (pharma-fMRI) has been considered an alternative or complimentary means of assessing the neural mechanisms of drug action (Wise and Tracey, 2006; Iannetti and Wise, 2007; Khalili-Mahani et al., 2017). Over the past decade, sophisticated pharma-fMRI methodologies have evolved for measuring hemodynamic changes by the BOLD signal or, in more advanced applications, by measuring cerebral blood volume (CBV) and arterial spin labeling (ASL) MRI sequences (Mandeville et al., 2001; Wang et al., 2011; Lu et al., 2014; see Jenkins, 2012) for review of the mechanism and applications of pharmacologic MRI, which includes a broader scope of the methodology than pharma-fMRI.

**Figure 1:**
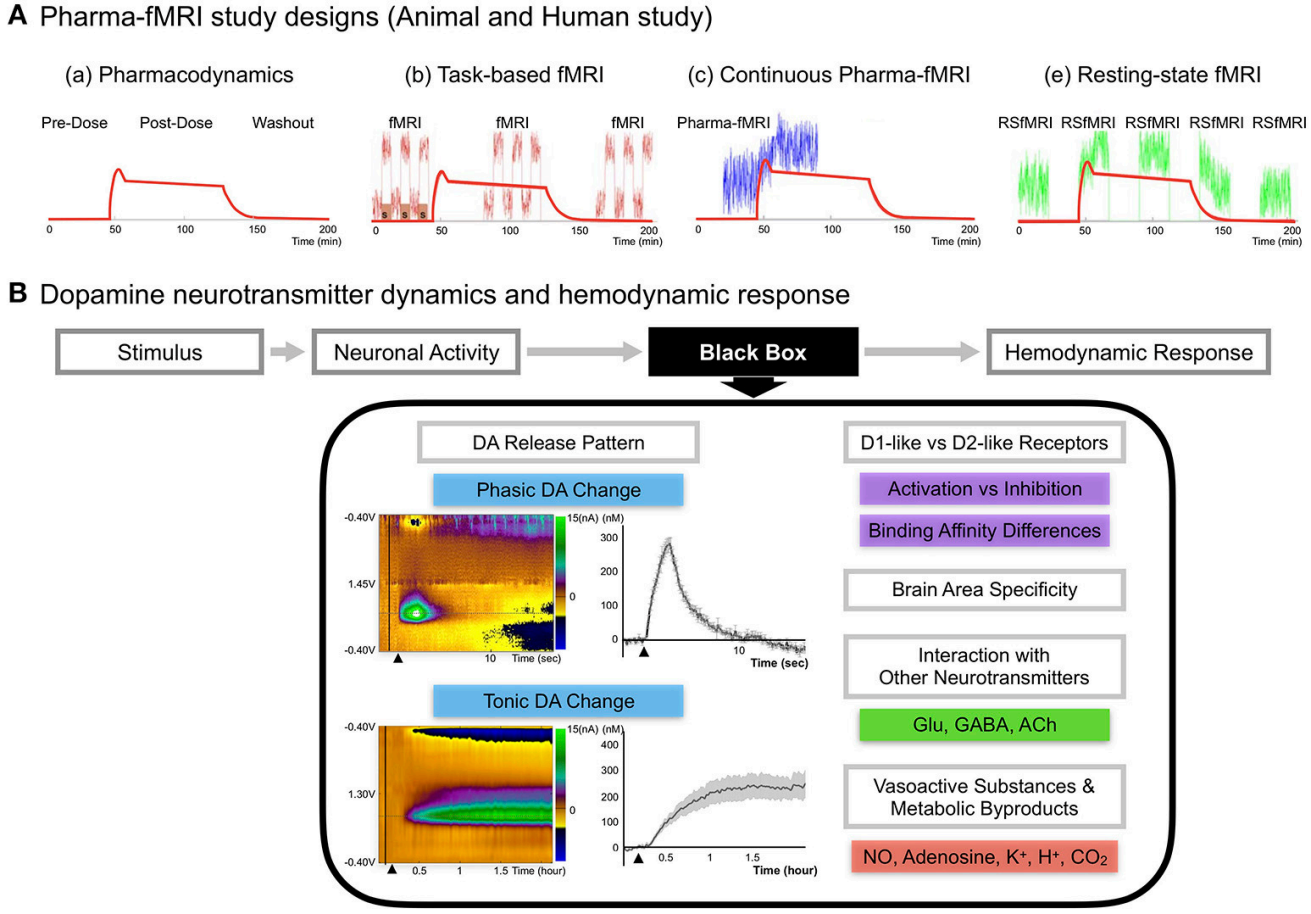
**(A)** Pharma-fMRI study designs. Pharma-fMRI is usually performed under three experimental designs: **(a)** The red line shows the pharmacodynamics model of a drug (morphine) (Khalili-Mahani et al., 2017). **(b)** Task-based pharma-fMRI analyzes a behavioral event at baseline and while the drug is under effect (Iannetti and Wise, 2007; Borsook et al., 2008); **(c)** continuous collection of fMRI over the course of drug infusion followed by analysis of the change in BOLD signal from pre-drug baseline (Bloom et al., 1999; De Simoni et al., 2013); and **(e)** pharmacological resting-state fMRI (pharma-RSfMRI) which examines several short resting-state intervals over the course of drug administration and compares network property changes across different phases of the pharmacokinetic profile (Khalili-Mahani et al., 2012; Lu and Stein, 2014). Diagram reprinted with permission from Khalili-Mahani et al. (2017). **(B)** DA neurotransmitter dynamics and hemodynamic response. A schematic of showing the possible factors affecting the link between DA neurotransmitter dynamics and the hemodynamic response (Concept adopted from Jenkins, 2012). Since there are no known voltage-gated vascular receptors, the mechanism by which neurotransmitter release and uptake leads to signaling and release of vasoactive molecules remains unknown, noted here as the “black box” (Jenkins, 2012). Recent updates on DA pathway mechanisms demonstrate a complex release pattern having both phasic and tonic states, dynamically modifying the basal tone of neuronal activity, as seen in disease or drug-induced states (Owesson-White et al., 2009; Grace, 2016). The phasic DA response in the upper left color map is from electrically stimulating (2 s) the nigrostriatal pathway and conducting neurochemical analysis in the caudate, which was confirmed by fMRI in a within subject study of a non-human primate (Min et al., 2016). Each electrochemical signal was converted to molar (M) concentration showing the difference from baseline to an electrically evoked response. The tonic DA response in the lower left color map is from systematic administration of nomifensine, a DA transporter reuptake blocker, and recording the electrochemical response in striatum over a time period of 2 h in rodents (Oh et al., 2016). Other factors are discussed in the current review, including DA receptor family type specific effects, brain area specificity, and DA integration with other neurotransmitters. Finally, neuronal activity would influence vasoactive substances (NO, K^+^) and metabolic by-products (Adenosine, H^+^, CO_2_) affecting the local hemodynamic response.

Several representative strengths of pharma-fMRI that complement PET include: (1) a relatively high spatial and temporal resolution; (2) elimination of the need for a radioactive tracer, making it less invasive and expensive than PET; and (3) increased flexibility in study design (Khalili-Mahani et al., 2017), including functional connectivity studies (Khalili-Mahani et al., 2012; Lu and Stein, 2014) (see Figure 1A).

There are numerous pharma-fMRI applications that are useful for studying the DA signal pathway, including DA cell lesion studies and receptor-type dependent agonist and antagonist studies (Chen et al., 1997; Takahashi et al., 2005). Recent updates on DA pathway mechanisms demonstrate a complex release pattern having both phasic and tonic states, dynamically modifying the basal tone of neuronal activity (Owesson-White et al., 2009; Grace, 2016; Oh et al., 2016). Disruption of this dynamic state of neuronal activity is a major mechanism of disease states and of neuropharmacological treatment regimens, which highlights the importance of studying neurotransmitter mechanisms when assessing hemodynamic changes (Grace, 2016). Thus, to properly interpret pharma-fMRI results, a more complete understanding of the relationship between the DA signaling pathway and the BOLD signal is needed. Figure 1B summarizes presently unexplained aspects of the relationship between fMRI/pharma-fMRI and electrophysiology, metabolism, and vasoactive molecule release (Jenkins, 2012).

## Dopamine neurotransmitter dynamics and hemodynamic response

Studies investigating DA release kinetics typically operate on the principle of targeting cell surface DA receptors (D1-like or D2-like) with a PET contrast agent tagged to competing ligands of interest. For example, Koepp et al. (1998) found that PET imaging of D2/D3 receptor occupancy following administration of ^11^C-raclopride, a selective D2/D3 antagonist, showed that binding of ^11^C-raclopride is significantly decreased in the brains of video game playing human subjects as a consequence of reward-induced DA release. Similarly, challenging endogenous DA with administered DA conjugated to PET contrast agents is a method of assessing task-induced changes in DA release in the human brain (Badgaiyan, 2014). While PET is sensitive to neurochemical activity and neurotransmission, it is limited by its temporal and spatial resolution. It fails to provide insights into functional correlates of neural activity associated with DA neurotransmitter dynamics since it is also influenced in a complex manner by the abundance of target receptors and the amount of neurotransmitter release.

Functional correlates of neural activity linked to DA release from reward areas in the brain have also been assessed in fMRI studies (Knutson et al., 2001). Studies in rats link DA release and either BOLD or CBV signals in temporal brain regions following stimulation with amphetamine (AMPH), which is known to induce the release of DA (Chen et al., 1997, 1999, 2005; Choi et al., 2006).

Several key factors may contribute to the observed coupling of DA release and the brain's hemodynamic response. The DA-mediated pharma-fMRI response can largely be explained in terms of receptor occupancies. For example, there is a positive correlation between DA displacements (PET) and fMRI in the ventral striatum and nucleus accumbens (NAc) in a behavioral task that evoked dopaminergic circuits from the substantia nigra (SN) and ventral tegmental area (VTA) (Pessiglione et al., 2006; Knutson and Gibbs, 2007; Schott et al., 2008). Lesion studies show that pharma-fMRI detects severe DA lesions in animals with sensitivity similar to that of PET (Chen et al., 1999; Schrantee and Reneman, 2014). Simultaneous PET/fMRI in non-human primate (NHP) enabled mapping of an association between DA release and neurovascular coupling (Sander et al., 2013). The potential for a temporal discrepancy between receptor occupancy and hemodynamic changes due to a phenomenon of receptor internalization and desensitization was found using the D2/D3 agonist quinpirole (Sander et al., 2016). Reports also show D3 agonist-induced CBV change to be a good match with D3 receptor mRNA expression (Choi et al., 2010). In another report, the administration of a graded dose of AMPH found that DA measured by microdialysis was linearly correlated with cyclic adenosine monophosphate (cAMP) levels as well as induced-CBV in the striatum of rats (Ren et al., 2009).

An important aspect of DA dynamics is the fact that D1-like family and D2-like family receptors have differing effects on activation and inhibition, respectively. Activation of the D1-like family receptors increases cAMP and induces excitatory signal transduction, and D2-like family receptors subsequently decrease cAMP, leading to an inhibitory effect in neurons (Schinelli et al., 1994). D3 receptor agonist, which is a D2-like category inhibitory receptor, shows negative changes in CBV, whereas the antagonist shows positive changes in CBV (Choi et al., 2010). This fact adds an additional layer of complexity to the DA-mediated hemodynamic response. For example, large increases in DA can inhibit the hemodynamic response. D1 receptors are responsible for an increase of hemodynamic response while D2 receptors are responsible for a decrease of hemodynamic response (Jenkins, 2012).

This relationship between the effects of receptor type (D1-like vs. D2-like) and hemodynamic response has been modeled by independently activating D1 and D2 receptors via cocaine, which is a DA transporter blocker, or via AMPH, which is an endogenous DA releaser (Mandeville et al., 2013). Studies, using AMPH and other pharmaceuticals to induce differential activation of DA receptors may assist in future work examining the intersection of DA and fMRI/pharma-fMRI BOLD responses. DA demonstrates a higher affinity for D2 receptors than D1 receptors (Richfield et al., 1989). D1 receptor activation is dominant in higher doses of AMPH, whereas the high affinity of DA for D2 receptors is expected to drive the response at lower doses. Negative CBV is evident with low doses of DA, but an increasingly higher positive CBV is observed with higher amounts of AMPH (Ren et al., 2009). There is also a species specific difference related to the D1 to D2 receptor ratio in that rats have a higher ratio of D1 to D2 receptors than NHP (Cumming, 2011) and thus exhibit higher positive CBV responses at high doses of DA inducing drugs than do NHP (Mandeville et al., 2011, 2013). Computational modeling studies add weight to these findings (Mandeville et al., 2013, 2014; Bruns et al., 2015). Receptor-based models of neurovascular coupling, incorporating receptor densities and affinities along with biophysical constraints, have demonstrated compelling descriptions of pharma-fMRI signals induced by dopaminergic stimuli (Mandeville et al., 2014). Mandeville et al. (2013) which confirms this model showing inhibition in fMRI at low dose of AMPH but biphasic response at higher doses in the basal ganglia of NHP (Mandeville et al., 2013).

Optogenetics is a technology that combines genetic and optical methods to control the excitatory or inhibitory pathway of targeted cells. Due to its high specificity and selectivity, optogenetics has been applied extensively to investigate functional circuitries in animal brains (Gradinaru et al., 2009; Lee, 2012). When applying DA cell specific optogenetic stimulation on VTA, DA neurons are activated, and the BOLD response occurs in both the VTA-innervated limbic regions, including the ventral striatum (NAc), and in non-VTA regions, including the dorsal striatum and the globus pallidus (Lohani et al., 2017). Another study conducting both direct electrical stimulation and optogenetic stimulation (targeting only DA cells) of the VTA have found to cause DA release in NAc, but only electrical stimulation has triggered significant BOLD responses in the medial prefrontal/anterior cingulate cortex and NAc (Helbing et al., 2016). Further glutaminergic N-methyl-D-aspartate receptor antagonist reduced the BOLD response by VTA electrical stimulation (Helbing et al., 2016). Given these discrepancies that exist depending on methods of stimulation in studies of DA-engendered BOLD responses, a novel framework to understand neurotransmitter-dependent BOLD functions is necessary. Such a framework could lead to innovative interpretations of the pharma-fMRI data obtained from human studies.

## Dopamine and neurovascular coupling

The fMRI signal cannot be interpreted exclusively in terms of neuronal activation. Neurovascular coupling, a process partially mediated by non-neuronal processes, must also be taken into account. Ultimately, hemodynamic changes are related to the release of vasoactive substances coupled to neurotransmitter activity, such as nitric oxide (NO), adenosine, and potassium cations (K^+^) (Attwell and Iadecola, 2002). In glutamatergic neurons, calcium influxes in postsynaptic neuron activities and in astrocyte activities during reuptake produce NO, adenosine and arachidonic acid metabolites (Attwell and Iadecola, 2002). In DA neurons, the uptake of DA occurs pre-synaptically and is not calcium dependent, although the DA release is, which implies that DA would not have the same neurovascular coupling as glutamate (Jenkins, 2012). While the fMRI signal through glutamatergic sensory stimulation could be blocked by NO synthase inhibition (Burke and Bührle, 2006), DA-evoked CBV through DA transporter blocker and D1 agonist was not sensitive to NO synthase inhibition (Choi et al., 2006).

DA neurons have been reported to directly innervate the intraparenchymal vessels in the cortex causing vasoconstriction as confirmed by histology and *in vitro* brain slices (Krimer et al., 1998). However, in the striatum, endogenous DA did not affect local blood flow, as the stimulation-evoked oxygen signal remains the same by DA synthesis blocker measured by fast-scan cyclic voltammetry (FSCV) (Zimmerman and Wightman, 1991).

Due to the ability of FSCV to simultaneously detect tissue diffused oxygen and extra-cellular DA responses in electrochemistry studies (Zimmerman and Wightman, 1991), correlations between extracellular oxygen and pH change have been reported to have a relationship with DA (Venton et al., 2003). Reports show that SN/VTA electrical stimulation evokes DA in the striatum as well as tissue diffused oxygen, which has two peaks (biphasic) within a 30 s timeframe as measured by millisecond temporal resolution of FSCV (Zimmerman and Wightman, 1991; Venton et al., 2003). Venton et al. (2003) further reports that NO and adenosine partially effect the first and second oxygen peak, respectively, independent of DA change (Venton et al., 2003). In addition both oxygen peaks are reduced by carbonic anhydrase inhibitor, which implies that the hemodynamic response for dopaminergic circuit stimulation relies on CO_2_ washout, which produces H^+^ as a byproduct (Huang et al., 1995).

## Multi-modal methodological approaches

Recent improvements in FSCV to correct for nonspecific signal drifting report reliable measurement of both phasic and tonic extracellular DA changes (Atcherley et al., 2013, 2015; Oh et al., 2016). This is important given the complexity of the relationship between DA neural dynamics and fMRI which is highlighted by reports showing an association between cell-firing rate and phasic/tonic DA concentration in extracellular space. In general, cell-firing rates can induce two distinct patterns of DA release. DA neurons in the midbrain fire at 1–5 Hz, which controls tonic, levels of DA that are capable of occupying high-affinity D2 receptors in terminal regions of the mesolimbic system. DA neurons firing at relatively high-frequency bursts (>20 Hz) lead to much larger amounts of phasic DA release that are capable of occupying low-affinity D1 receptors in a short time period (Grace, 1991; Phillips et al., 2003; Dreyer et al., 2010). Typically, bursting activity of DA neurons over brief periods (<300 ms) is associated with reward mechanisms in NHP under the modulation of glutamatergic and cholinergic inputs (Gronier and Rasmussen, 1998; Schultz, 1998; Kitai et al., 1999). Tonic levels of DA are modulated by presynaptic limbic and cortical glutamatergic inputs in addition to DA neuronal signals (Grace, 1991; Howland et al., 2002).

Implanting electrical, optogenetic, or drug infusion probes through stereotactic surgery and measuring the local phasic and tonic DA response by FSCV and the global response by fMRI offers a unique combination of modalities to tease apart pre- and post-synaptic-specific effects. At present, few fMRI studies have addressed this issue. However, as in electrophysiology recordings, FSCV recordings require implanting a sensing electrode into brain tissue and probing to get a signal, sometimes referred to as the DA-hotspot in striatum, which connects to a specific DA circuit or behavioral task (McCutcheon et al., 2012; Kruss et al., 2017). Moreover, the basal ganglia and striatum have functional territories related to motor, limbic, and associative functions (Graybiel et al., 1994; Krack et al., 2010; Min et al., 2012), which makes the specificity of stimulation site and recording site all the more important (Kringelbach et al., 2007; Da Cunha et al., 2015). To identify the DA-specific recording site for FSCV in NHP, Min et al. (2016) used a combination of FSCV and fMRI, electrically stimulating the nigrostriatal pathway and measuring global response using fMRI (Min et al., 2016). Using the fMRI responses in caudate and putamen evoked by electrical stimulation of the subthalamic nucleus, they found that fMRI can be used to identify DA-specific FSCV recording sites. Similarly Settell et al. (2017) used fMRI BOLD responses in the NAc while stimulating VTA to confirm neurochemical recording of DA in NAc (Settell et al., 2017).

**Figure 2:**
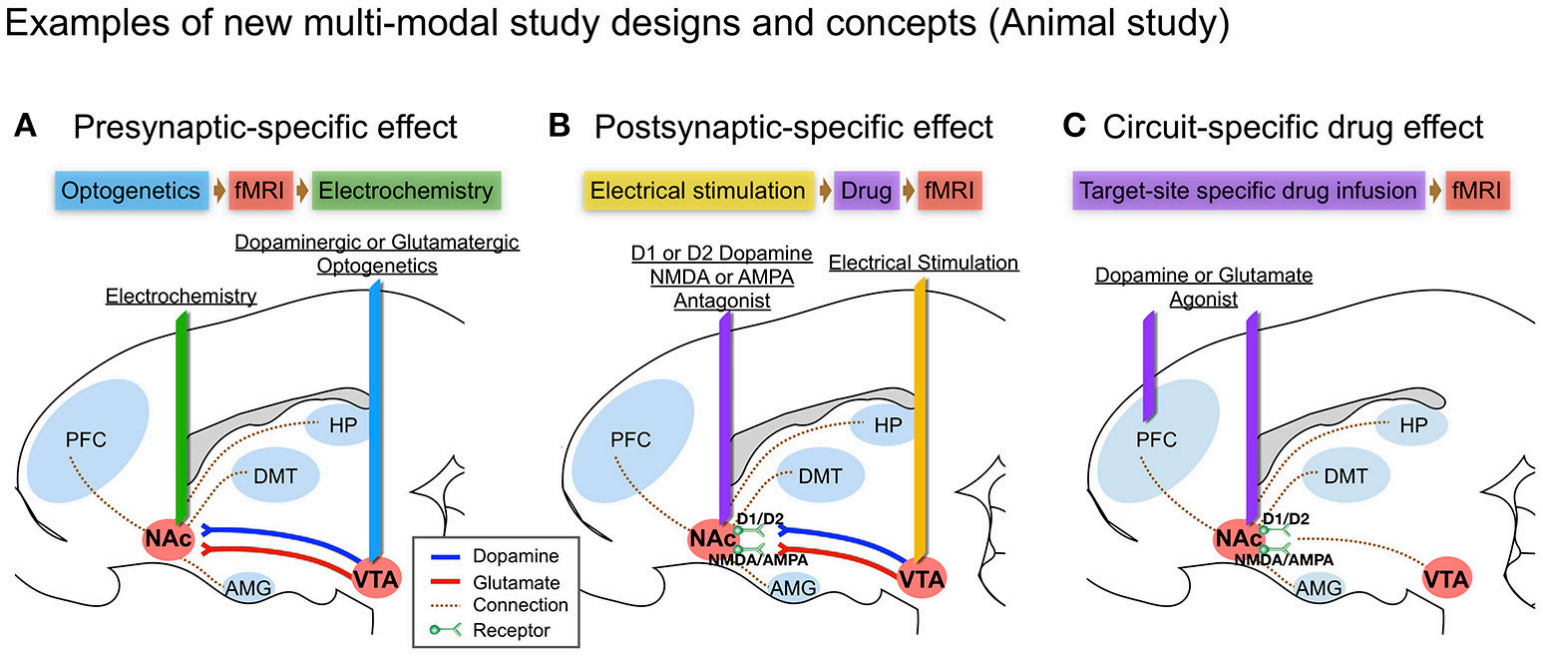
Examples of new multi-modal study designs and concepts in large animal (swine) brain diagram: **(A)** Presynaptic-specific effect. Optogenetics enables cell-type specific stimulation, and the fMRI signal induced by stimulation would provide insight into presynaptic-specific effects on BOLD (Albaugh et al., 2016; Helbing et al., 2016; Lohani et al., 2017) along with electrochemical information; **(B)** Postsynaptic-specific effect. Electrical stimulation of a specific pathway forces non-specific activity to the neuronal circuit involving all cell-types near the electrode (Kringelbach et al., 2007), combined with electrode-metal induced susceptibility artifact corrected fMRI (In et al., 2017). Administering receptor antagonists along the stimulated pathway enables the evaluation of receptor-specific effects on BOLD (Ross et al., 2016); **(C)** Circuit-specific drug effect. Popular in animal studies, brain circuit-specific intracranial drug injection (Wise and Hoffman, 1992; Kim et al., 2014) combined with fMRI, could open new possibilities for studying circuit-specific neurotransmitter (agonist) effects on BOLD by limiting the involvement of presynaptic dynamics.

Optogenetic stimulation evoked DA in specific brain circuits has also been measured by FSCV (Bass et al., 2010; Adamantidis et al., 2011), and there are other reports of combining optogenetic stimulation with fMRI (Gradinaru et al., 2009; Lee, 2012), but to our knowledge, there are no reports of combining optogenetic stimulation with both FSCV and fMRI. Of note, several studies suggest that optogenetic and electrical stimulation of an identical structure can cause fundamentally different neuronal activation patterns as measured by BOLD (Ohayon et al., 2013; Albaugh et al., 2016). The combination of fMRI and FSCV to measure circuit specific stimulation via optogenetic and via electrical stimulation would provide new insights, especially given that optogenetic stimulation may generate greater pre-synaptic selectivity than electrical stimulation (see Figure 2A).

Intracranial micro-infusions is another often used means of causing brain circuit-specific drug effect (Wise and Hoffman, 1992). Since DA receptors can also be found in the pancreas, kidneys, and blood vessels outside the brain, DA could, for example, cause vasodilation in the heart (Missale et al., 1998). Thus, intracranial micro-infusion could avoid such systemic blood flow changes that could influence fMRI results. Intracranial micro-infusions of a specific receptor antagonist (i.e., D1-like or D2-like) at specific brain sites during a stimulation-induced signaling cascade could be used to discriminate receptor-type specific post-synaptic effects (See Figure 2B). Using micro-infusion and BOLD fMRI has been reported in a swine model, showing that Fornix stimulation transmits a dopaminergic and glutaminergic specific response through NAc, affecting prefrontal cortex, amygdala and hippocampus (Ross et al., 2016). Borland and Michael (2004) also showed a decrease of tonic DA release in the rat striatum by intrastriatal infusion of kynurenic acid, a broad-spectrum antagonist of the ionotropic glutamate receptors (Borland and Michael, 2004). There is also a recent report of chronic adaptation to an intracranial micro-infusion drug system that could be implanted through stereotactic surgery in the large animal brain (swine) (Kim et al., 2014). Micro-infusion-induced DA changes (i.e., through DA agonist) and related changes in the BOLD signal can be used together to identify brain site- and neurotransmitter-specific neuronal activity (see Figure 2C).

The use of nanoparticles to more directly detect DA is a new approach on the horizon (Lee et al., 2014). Nanoparticle contrast agents that generate signals based on interactions with endogenously released DA have been introduced for MRI (Kim et al., 2014). The MRI intensity can be modulated based on magnetic field strength or certain properties of the reporter agent such as proton relaxivity and the chemical exchange saturation transfer effect. Bioresponsive contrast agents can be synthesized by engineered metalloproteins or by creating artificial host receptors, which derive from principles of supramolecular host-guest chemistry (Angelovski and Tóth, 2017).

## Summary and future directions

As fMRI and pharma-fMRI continue to expand in application, there is increasing need for a better understanding of the mechanisms by which neurochemical changes induced by pharmacologic agents affect the BOLD signal. The complexity of neurotransmitter dynamics requires sophisticated experimental designs. Several emerging methodologies that enable selective modulation of the presynaptic and postsynaptic pathways relative to neurotransmitter and receptor function detection and manipulation have been reviewed here. By examining these effects through the lens of DA dynamics, studies would be better positioned to address drug-induced neuronal and neurochemical changes relative to the BOLD signal. While each of the methodologies mentioned are readily available, future research is needed to expand the utility of combining these technologies so as to make multi-modality platforms more accessible in investigations of the effects of pharmacological agents on the BOLD signal in pharma-fMRI.

## Author contributions

Designed framework and idea: HJ and H-KM. Drafted and critically reviewed the manuscript: TB, VS, YO, DJ, S-YC, GW, VL, HJ, H-KM.

### Conflict of interest statement

The authors declare that the research was conducted in the absence of any commercial or financial relationships that could be construed as a potential conflict of interest.
